# Supplementary material for: Self-sorting in macroscopic supramolecular self-assembly via additive effects of capillary and magnetic forces
Source: Nat Commun. 2022 Sep 3;13:5201. doi: 10.1038/s41467-022-32892-y (PMC9440903; doi:10.1038/s41467-022-32892-y)
Supplement: Supplementary file 1 — Supplementary Information [file 41467_2022_32892_MOESM1_ESM.pdf]

Supplementary Information

Self-sorting in macroscopic supramolecular self-assembly via additive effects of capillary and magnetic forces

Tan et al

This Supplementary Information document includes:

Supplementary Note 1: Materials and Instrument 3

Supplementary Note 2: Fabrication of MSA Building Blocks 4

Supplementary Note 3: Surface Modification of EPS Building Blocks.............................. 6

Supplementary Note 4: Derivation of Contour Functions of Menisci 8

Supplementary Note 5: Measurements and Calculations of Capillary Forces.................. 8

Supplementary Note 6: Measurement/Simulation of Magnetic Forces/Field.................. 10

Supplementary Note 7: Time consumption to complete selective ABA assembly 11

Supplementary Note 8: Global Effects of Magnetic Field Leading to Isomers................ 12

Supplementary References.. 12

Supplementary Figures 1–9 below corresponding notes.

Supplementary Note 1: Materials and Instrument

Materials

The following chemicals were used as purchased: poly(ethyleneimine) (PEI) (branched, Mw 1800), poly(acrylic acid) (PAA) (aq, 25 wt%, Mw 240000), and 1H, 1H, 2H, 2H-perfluorooctyltrichlorosilane from Alfa Aesar; poly(diallyldimethylammonium chloride) (PDDA) (aq, 20 wt%, Mw 400000) from Sigma-Aldrich; poly(sodium-p-styrenesulfonate) (PSS) (Mw 70000) from Acros Organics; polydimethylsiloxane (PDMS) silicone elastomer (Sylgard 184) from Dow Corning; thioglycolic acid from Tokyo Chemical Industry; silicon dioxide and tetrachloroauric acid (50%) from Shanghai Macklin Biochemical Co., Ltd; chloroplatinic acid hexahydrate from Shanghai Aladdin Bio-Chem Technology Co., Ltd; hydrogen peroxide solution (aq, 30 wt%), dyes, n-hexane, and other normal chemical reagents from Sinopharm Chemical Reagent Beijing Co., Ltd. Expanded polystyrene, glass slides sputtered with gold, and soft rubber magnetic strips (permanent magnets) were commercially available.

Instruments

PDMS surface was pre-treated with oxygen plasma (ZEPTO, Diener electronic, Germany). Electrochemical deposition of gold and platinum aggregates was conducted with an electrochemical workstation (CHI600E, CH Instruments, China). Morphology and elemental composition of gold and platinum were characterized with scanning electron microscopy (SEM) and energy dispersive spectrometer (EDS) (Supra55, Zeiss Microscopy, Germany). The magnetic strength of magnetic plates was measured with a gaussmeter (WT106, Weite Magnetic Technology Co., Ltd, China). Force measurements were conducted on a dynamic contact angle measuring device and tension meter (DCAT21) and water contact angle (WCA) were measured on an OCA20 instrument, both of which were from Data Physics Instruments GmbH (Filderstadt, Germany). Optical photographs and videos were taken with a Nikon camera (D7000, Japan).

Supplementary Note 2: Fabrication of MSA Building Blocks

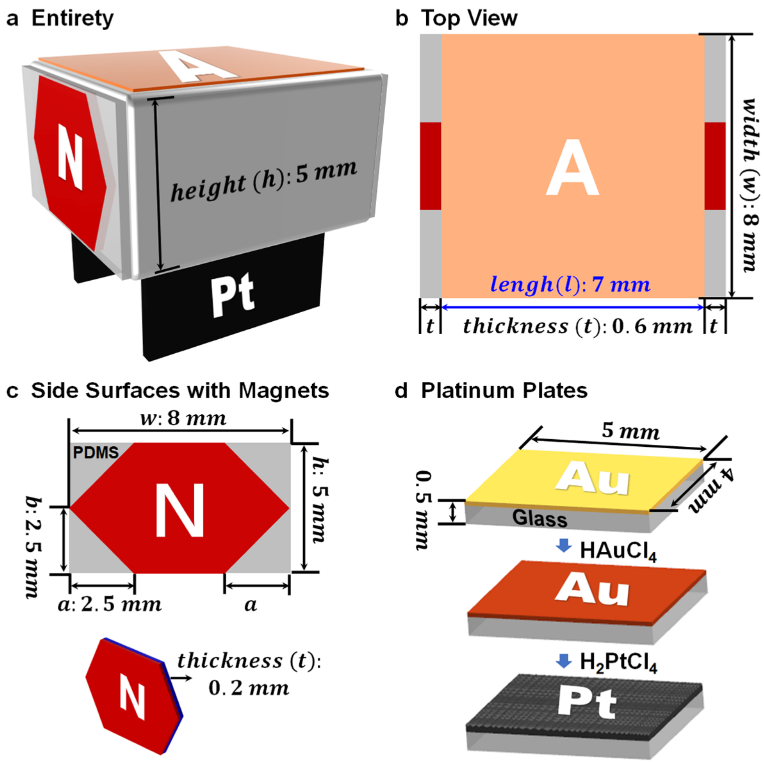

Supplementary Figure 1. Schematic illustration of MSA building block based on a cuboid EPS. a, The entire structure and **b**, the top view of A building block marked with size details; **c**, the size of magnets embedded in PDMS, which were further adhered onto two opposite side surfaces of A; **d**, the fabrication procedure of rough platinum plates to be inserted to the bottom of the building block.

Structural details of MSA building blocks

As shown in **Supplementary Figure 1**, the main part is a grey cuboid of expanded polystyrene (EPS) with a dimension of 8 mm × 7 mm × 5 mm. Two opposite side surfaces of EPS were adhered with two permanent magnets embedded in thin PDMS plates with a dimension of 8 mm × 5 mm × 0.6 mm (**Supplementary Figure 1b**); the magnetic plates were pre-cut in a hexagonal shape with a thickness of 0.2 mm (**Supplementary Figure 1c**). Two glass slides deposited with platinum aggregates (noted as Pt plates shown in **Supplementary Figure 1a**) were symmetrically inserted to the bottom surface of EPS with an orientation of being parallel to the two side surfaces without magnets. These two Pt plates have a dimension of 5 mm × 4 mm × 0.5 mm.

Fabrication of platinum aggregates

The above Pt plates were prepared by a two-step electrochemical deposition (**Supplementary Figure 1d**) according to a previous report[1]. Firstly the glass slides sputtered with gold was pre-treated by immersing in a solution of thioglycolic acid (ethanol, 20 mM) for 2 h; secondly electrochemical deposition was conducted in a mixed solution of H_2SO_4 (aq, 0.5 mM) and $HAuCl_4$ (aq, 1 mg/mL) for 1600 s (-200 mV) by using the above slides, a Ag/AgCl, and a Pt electrolde as the working, reference, and counter electrodes, respectively; afterwards, the plates were rinsed with deionized water and dried in nitrogen flows; thirdly the above plates were conducted with a second deposition following the same mode except changing to a mixed solution of H_2SO_4 (aq, 0.5 mM) and H_2PtCl_6 (aq, 1 mg/mL) for 2400 s (-200 mV), followed by rinsing and drying.

The morphology and elemental composition were characterized with SEM and EDS as shown in **Supplementary Figures 2a-b**. The presence of Au and Pt elements has confirmed the components of the deposited aggregates; the displayed rough structures of platinum are favorable for the catalyzed decomposition of H_2O_2 solutions to release oxygen bubbles, which provided the driving forces for the self-propulsion of MSA building blocks.

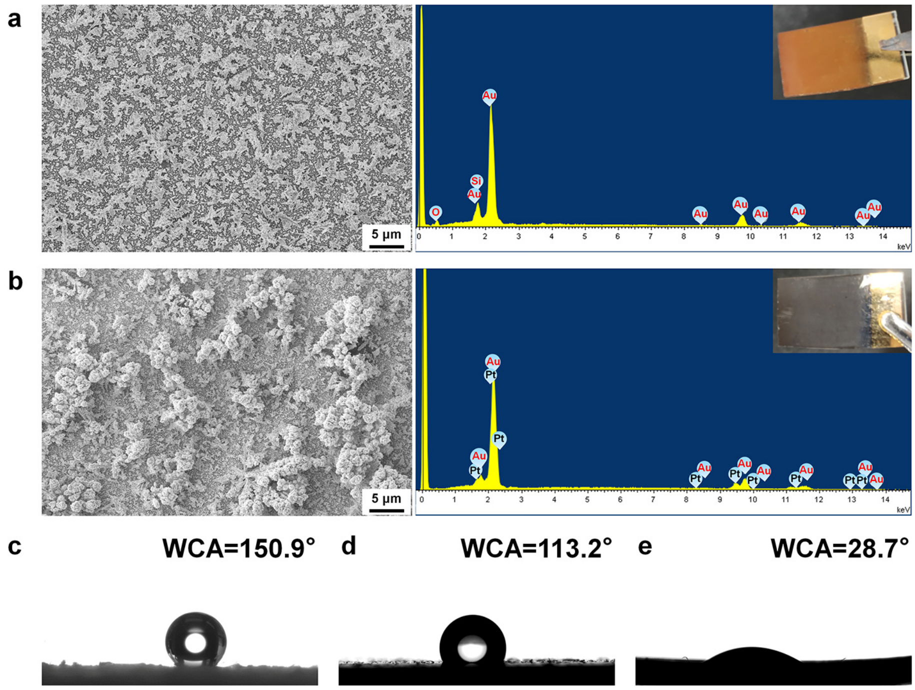

Supplementary Figure 2. Surface morphology and wettability of the main components of the MSA building blocks. SEM images and EDS patterns of the glass slide surfaces after the electrochemical deposition in **a**, $HAuCl_4$ and in **b**, H_2PtCl_6; the insets

are the optical photographs of the plates. WCA images of **c**, EPS, and PDMS embedded with magnetic plates **d**, before and **e**, after the surface modification of polyelectrolyte multilayers.

Supplementary Note 3: Surface Modification of EPS Building Blocks

Modification of hydrophobic side surfaces of EPS

According to a reported method[2], we have prepared a solution for the surface modification to obtain superhydrophobicity: (1) 1.0 g silicon dioxide (15 ± 5 nm) were dispersed in hexane and stirred for 3 h; (2) 1 mL of 1H, 1H, 2H, 2H-perfluorooctyltrichlorosilane was added and the resulted mixture was further stirred for 8 h, followed by settlement for half an hour; (3) after removing the supernatant, the solid was washed with ethanol for 3 times and dried at 60 °C; (4) the dried solid was dispersed in ethanol with a weight/volume ratio of 4 mg/mL. The purchased EPS cuboids were immersed in the dispersion for 5 min and dried in air at room temperature. The resulted water contact angle (WCA) of the modified EPS surface was 150.9° (**Supplementary Figure 2c**), proving the successful modification of superhydrophobicity.

Modification of hydrophilic side surfaces of EPS

The permanent magnets were embedded in PDMS by a sandwiched molding method (**Supplementary Figure 3a**); afterwards the entirety was modified with polyelectrolyte multilayers by a layer-by-layer (LbL) self-assembled technique (**Supplementary Figure 3b**). Firstly, the soft magnetic strips were cut into a hexagonal shape with a dimension shown in **Supplementary Figure 1c** and then aligned on a normal glass substrate, the four corners of which were adhered with four coverslips (thickness: ~0.6 mm) as spacers. Secondly, a precursor mixture of PDMS containing pre-polymer, crosslinker and dye with a mass ratio of 10:1:0.1 were poured onto the aligned magnets. To distinguish the surface chemistry of PDMS for the subsequent LbL processes, we have dyed A building blocks as red and B building blocks as green in the PDMS precursor liquid. Thirdly, we covered these PDMS and magnets with another glass to form a sandwich structure, followed by heating at 65 °C for 4 h to crosslink PDMS. Finally, after de-molding we cut the PDMS into a dimension of 8 mm $\times$ 5 mm $\times$ 0.6 mm with the magnet embedded in the center. The

magnetic strength on the surface was measured to be about 5 mT with a gaussmeter. Normal PDMS plates without magnets were prepared following the same procedure except for the step of adding magnets.

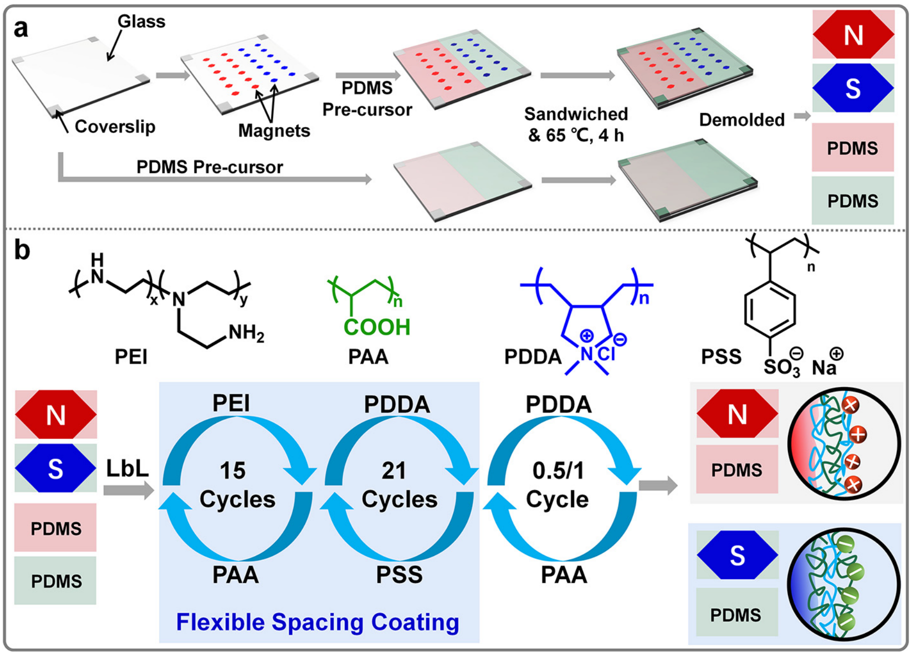

Supplementary Figure 3. Fabrication of PDMS with and without magnets and the surface modification. a, Sandwiched molding method. **b**, Surface modification of as-prepared PDMS with and without magnets via LbL technique with shown polyelectrolytes.

The surface modification of the above PDMS or PDMS embedded with magnets was realized by LbL processes: (1) they were cleaned by ultrasonication in ethanol for 10 min, dried with nitrogen flows, and treated with oxygen plasma for 3 min to obtain a hydrophilic surface; (2) the treated plates were immersed a PEI solution (aq, 1 mg/mL) for 6 h, followed by alternate immersion in a PAA solution (aq, 1 mg/mL) and PEI for 1 min each, between which the plates were rinsed with deionized water; after 15 alternate cycles of PEI-PAA, we obtained a polyelectrolyte multilayer noted as $(PEI/PAA)_{15}$; (3) subsequently, the plates were alternately immersed in PDDA (aq, 1 mg/ml) and PSS (aq, 1 mg/ml) solutions for 5 min each until 21 cycles to obtain a $(PEI/PAA)_{15}$-$(PDDA/PSS)_{21}$ multilayer, which is known as the 'flexible spacing coating' to facilitate macroscopic adhesion[3]; (4) the outmost positive or negative charges were induced by immersing in PDDA (aq, 1 mg/ml) for 5 min or conducting one LbL cycle in PDDA and PAA (aq, 1 mg/ml) solutions for 5 min each. Taken together, PDMS embedded with magnets were modified with two kinds of surface

chemistry: one is $(PEI/PAA)_{15}$-$(PDDA/PSS)_{21}$-PDDA and the other is $(PEI/PAA)_{15}$-$(PDDA/PSS)_{21}$-PDDA/PAA, which were further adhered onto the opposite side surfaces of A building blocks and B building blocks, respectively, to induce both hydrophilic surface wettability and interactive groups of positive or negative charges. As shown in **Supplementary Figures 2d-e**, the WCA values of PDMS surfaces were measured to be 113.2° and 28.7° before and after LbL modification, having confirmed the transition from hydrophobic to hydrophilic.

In all experiments with magnetic forces, building blocks have north poles of magnets facing outward were marked as 'A' while building blocks have south poles facing outward were marked as 'B'. In control experiments without magnetic forces (**Figure 4b**), normal PDMS plates without embedding any magnets were modified similarly following the above LbL processes to obtain hydrophilic surfaces. In control experiments of 'all-hydrophilic' side surfaces (**Figures 5d-e**), the commercially available EPS were modified to be hydrophilic with the above LbL processes.

Supplementary Note 4: Derivation of Contour Functions of Menisci

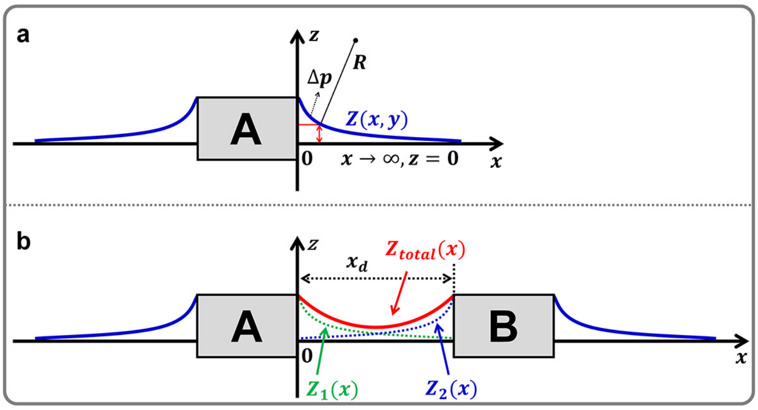

Supplementary Figure 4. Force analysis of menisci. a, one and **b**, two MSA building blocks at the air/water interface. The blue and red lines indicate the contours of the menisci of hydrophilic side surfaces.

Supplementary Note 5: Measurements and Calculations of Capillary Forces

The capillary attraction was quantified by the following steps with hydrophilic side surfaces as an example. *(1) Measurement of the critical distance to trigger capillary attraction of building blocks.* We fixed A building block and released B building block at different

positions of varied distance (x_d) from A (**Supplementary Figure 5a**). If A is located within the interactive distance of capillary forces, we observed fast movements of A towards B; otherwise, A kept static where we placed. As we changed the distance (x_d=20, 21, 22, 23, 24, and 25 mm), we observed the movements of A at x_d=22 mm, but A remained static at x_d=23 mm, indicating a roughly critical distance of the capillary interaction to be 22 mm in our assembly system (**Supplementary Figure 5b**). *(2) Calculation of the instantaneous force based on the motion trajectories of A in the above capillary attraction.* We took a video to record the above capillary attraction between A and B and showed some snapshots in **Supplementary Figure 5a**. We measured the distance that A had moved after a certain time interval, e.g., s_t at t=t_2; based on Newton's law of motion, we calculated the instantaneous moving velocity (v_t) and the acceleration (a_t) at this moment; by neglecting the fluidic friction due to the low Reynold number, we roughly obtained the attractive force for the assembly, namely, the lateral capillary force (F_{MSA}) shown in **Figure 3a**. The results of capillary forces obtained by the measurements of the critical interactive distance and the simulation via contour the functions have been co-plotted in **Supplementary Figure 5c**. The results matched well with each other when the two building blocks were away from a distance larger than 8 mm, but some deviation appears when they approached closer; the dramatically increasing trends of the forces values with the decreasing distance remain the same in both cases. The deviation may be caused by slight rotations (shown with arrows in **Supplementary Figure 5a**) contributed by wettability conflicts, which was not considered in the simulation due to model simplification and calculation. Theoretically, the capillary forces from both hydrophilic and hydrophobic side surfaces increase dramatically when the two building blocks approach into proximity, which increases the driving force together and favors for long-ranged alignment to realize precise matching between the interactive surfaces. Besides, complex fluidic dynamics may also influence the motions.

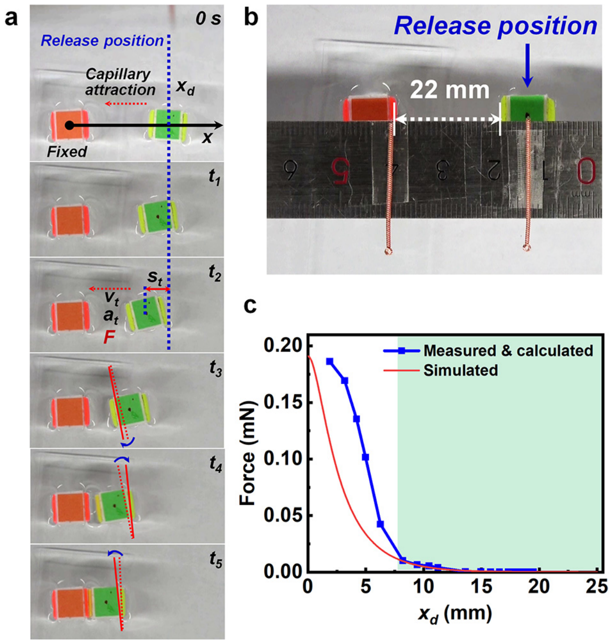

Supplementary Figure 5. Measurement and calculation of capillary attraction. a, Snapshots of top view when two MSA building blocks approached due to the capillary attraction. Blue arrows indicate slight rotations. **b**, Photo of building blocks with the critical interactive distance (22 mm). **c**, Capillary forces obtained by the measurements of critical interactive distance (blue line) and the simulation based on contour functions (red line). Source data are provided as a Source Data file.

Supplementary Note 6: Measurement/Simulation of Magnetic Forces/Field

As shown in **Supplementary Figure 6a**, we applied a force apparatus of DCAT21 to measure the magnetic force dependent on the distance between the magnetic plates used in experiments. The force apparatus has a 'reserve scale' installed on the top to indicate mass changes on the sensor. We connected one magnetic plate to the sensor with a thread and fixed the other magnetic plate on the motor at the bottom; their N and S poles are placed opposing each other. As schematically illustrated in the insets (I-V) of **Supplementary Figure 6b**, the measurement was conducted by a closed loop of 'approach-separation' between the magnetic plates. The motor moved upward to make the fixed magnetic plate approach the pensile magnetic plate and then moved downward to result in the separation of the two magnetic plates. Accordingly, the mass changes on the sensor were recorded as a mass-position curve shown in **Supplementary Figure 6b**; the peak value of the curve in the approach process (blue line) indicated the maximum

magnetic attraction, which occurred when the two magnets contact each other, namely their interactive distance (x_d) was zero. After calculations of converting the mass changes into the force changes and calibrating the interactive distance, we obtained the measured magnetic forces versus the interactive distance between MSA building blocks (**Figure 3**). The measurement of magnetic repulsion is difficult with this apparatus because the pensile magnet drifted with the N-N repulsion. Considering that the magnetic attraction and repulsion only has an opposite direction while other parameters are similar, we took the values of the obtained magnetic attraction to indicate the contribution of magnetic repulsion (**Figure 3c**). The secondary magnetic force ($F_{mag-N-2S}$) shown in **Figure 5a** was measured with this method.

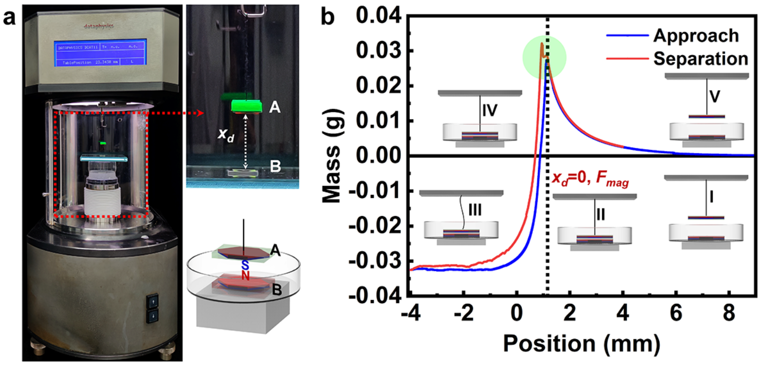

Supplementary Figure 6. Measurements of magnetic forces. a, Photograph of the force apparatus and schematic illustration of locally magnified magnetic plates under measurements. **b**, Mass-position curve recorded in the approach-separation processes between two magnetic plates. Source data are provided as a Source Data file.

Supplementary Note 7: Time consumption to complete selective ABA assembly

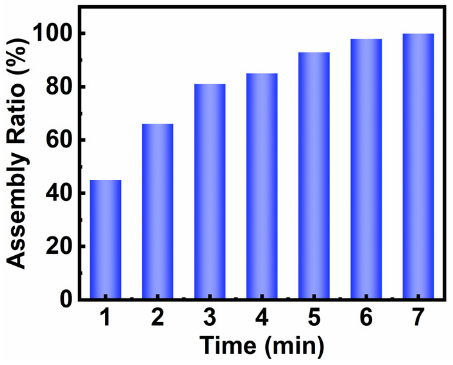

Supplementary Figure 7. Time-dependent assembly ratio of 100 parallel assembly events of ABA structures shown in Fig. 4a. Source data are provided as a Source Data file.

The time to realize selective ABA assembly shown in Fig. 4a has been summarized in **Supplementary Figure 7**. Over 50% groups realized selective and precise assembly in 2 min and the left ones were all assembled in totally 7 min.

Supplementary Note 8: Global Effects of Magnetic Field Leading to Isomers

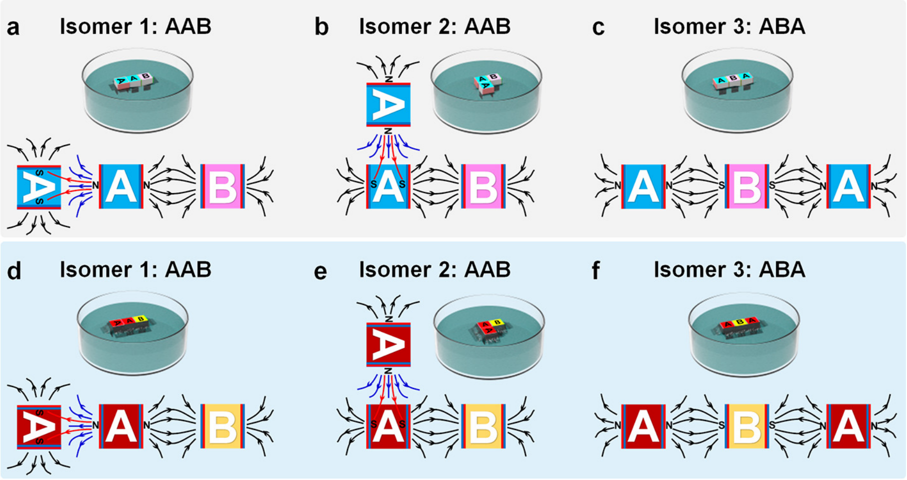

Supplementary Figure 8. Schematic illustration of isomers of trimers in MSA. All side surfaces are **a-c**, hydrophobic or **d-f**, hydrophilic.

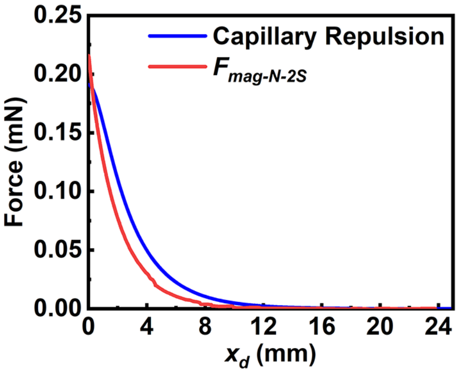

Supplementary Figure 9. Force-distance curves of the secondary magnetic attraction ($F_{mag-N-2S}$) and the capillary repulsion between menisci of opposite shapes. Source data are provided as a Source Data file.

Supplementary References

1. Cheng, M., Ju, G., Zhang, Y., Song, M., Zhang, Y. & Shi, F. Supramolecular assembly of macroscopic building blocks through self-propelled locomotion by dissipating chemical energy. *Small* **10,** 3907-3911 (2014).
2. Tian, P., Gao, X., Wen, G., Zhong, L., Wang, Z. & Guo, Z. Diving-floating locomotion induced by capturing and manipulating bubbles in an aqueous environment. *Chem. Commun.* **54,**

11713-11716 (2018).

3. Cheng, M., Shi, F., Li, J., Lin, Z., Jiang, C., Xiao, M., Zhang, L., Yang, W. & Nishi, T. Macroscopic supramolecular assembly of rigid building blocks through a flexible spacing coating. *Adv. Mater.* **26,** 3009-3013 (2014).
