# Supplementary material for: Self-sorting in macroscopic supramolecular self-assembly via additive effects of capillary and magnetic forces
Source: Nat Commun. 2022 Sep 3;13:5201. doi: 10.1038/s41467-022-32892-y (PMC9440903; doi:10.1038/s41467-022-32892-y)
Supplement: Supplementary file 3 — Description of Additional Supplementary Files [file 41467_2022_32892_MOESM3_ESM.pdf]

File name: Supplementary Movie 1

Description: The as-prepared A building block with a density of about 0.47 g/cm^3 was placed onto an aqueous solution of H_2O_2 (10 wt%, density: 1.048 g/mL). The side surfaces of the building block were alternately modified as hydrophilic and hydrophobic to induce wettability conflicts. The vigorously released oxygen bubbles exerted asymmetric forces to result in the self-propulsion of the building blocks and the irregular motion trajectory.

File name: Supplementary Movie 2

Description: Two as-prepared building blocks were placed onto an aqueous solution of H_2O_2 (10 wt%, density: 1.048 g/mL) for MSA. For the red-colored one (noted as A in the text), the PDMS plates embedded with magnets were modified with positive charges based on a composite polyelectrolyte multilayer of $(PEI/PAA)_{15}$- $(PDDA/PSS)_{21}$-PDDA; the north poles of the magnets were placed towards outward. For the green-colored one (noted as B in the text), the PDMS plates had negative charges on the surfaces base on a $(PEI/PAA)_{15}$- $(PDDA/PSS)_{21}$-PDDA/PAA multilayer. Both A and B underwent self-propulsion and they assembled to a precisely matched dimer in the end owing to the additive effects of magnetic/capillary interactions. After the assembly, the dimer could be immediately lifted out of the air/water interface, indicating the rapid and stable electrostatic interactions between the assembled surfaces.

File name: Supplementary Movie 3

Description: Two A building blocks in red color were placed onto an aqueous solution of H_2O_2 (10 wt%, density: 1.048 g/mL). The PDMS plates attached onto their side surfaces were modified with a composite polyelectrolyte multilayer of $(PEI/PAA)_{15}$-$(PDDA/PSS)_{21}$-PDDA; the north poles of the magnets were placed towards outward. Both A and B underwent self-propulsive motions and they repel each other upon approaching into proximity because of the competitive interactions of magnetic repulsion and capillary attraction. The N-N repulsion is dominant to result in the repulsion.

File name: Supplementary Movie 4

Description: The assembly of the ABA trimer was conducted on an aqueous solution of H_2O_2 (10 wt%, density: 1.048 g/mL). Two A building blocks in a red color had two opposite side surfaces modified with a $(PEI/PAA)_{15}$-$(PDDA/PSS)_{21}$- PDDA multilayer; the north poles of the magnets were placed towards outward. One B building blocks in a green color had two opposite side surfaces modified with a $(PEI/PAA)_{15}$-$(PDDA/PSS)_{21}$-PDDA/PAA multilayer; the south poles of the magnets were placed towards outward. The three building blocks underwent self-propulsion and formed an ABA trimer owing to the self-soring mechanism in the presence of the magnetic/capillary addition.

File name: Supplementary Movie 5

Description: Control experiments of two isomers in the assembly of trimer structures were conducted on an aqueous solution of H_2O_2 (10 wt%, density: 1.048 g/mL). Two A building blocks in a red color had two opposite side surfaces modified with a $(PEI/PAA)_{15}$-$(PDDA/PSS)_{21}$- PDDA multilayer; one B building blocks in a green color had two opposite side surfaces modified with a $(PEI/PAA)_{15}$-$(PDDA/PSS)_{21}$-PDDA/PAA multilayer. No magnets were added to A or B. The three building blocks exhibited self-propulsive motions but formed two kinds of isomers: (1) an ABA trimer and (2) an AAB trimer. In the

absence of magnetic sorting, the capillary-driven MSA could not identify the A-B or A-A assembly, thus leading to the formations of isomers.

File name: Supplementary Movie 6

Description: The application of the self-sorting mechanism to fabricate advanced structures were conducted on an aqueous solution of H_2O_2 (10 wt%, density: 1.048 g/mL). All A building blocks in a red color had two opposite side surfaces modified with a $(PEI/PAA)_{15}$-$(PDDA/PSS)_{21}$- PDDA multilayer; the north poles of the magnets were placed towards outward. All B building blocks in a green color had two opposite side surfaces modified with a $(PEI/PAA)_{15}$-$(PDDA/PSS)_{21}$-PDDA/PAA multilayer; the south poles of the magnets were placed towards outward. After self-propulsions to approach, the building blocks formed a tetramer, a hexamer, and an octamer in the end.

File name: Supplementary Movie 7

Description: Control experiments in the absence of wettability conflicts were conducted on an aqueous solution of H_2O_2 (10 wt%, density: 1.048 g/mL). All the side surfaces of A or B were modified hydrophobic with a coating of silicon dioxide nanoparticles (15 ± 5 nm) that were pre-modified with 1H, 1H, 2H, 2H-perfluorooctyltrichlorosilane. The difference is that A has north poles of magnets facing outward while B has south poles facing outward. After self-propulsive motions, three isomers of ABA, triangled AAB, and lined AAB were observed. The secondary magnetic force of ($F_{mag-N-2S}$) between one N pole of one A and two S poles of another A that was rotated by 90°, resulted in the isomers of AAB; the absence of local capillary repulsion based on the design of wettability conflicts between adjacent side surfaces, was also a reason for these phenomena.

File name: Supplementary Movie 8

Description: Control experiments in the absence of wettability conflicts were conducted on an aqueous solution of H_2O_2 (10 wt%, density: 1.048 g/mL). All the side surfaces of A or B were modified hydrophilic but with different surface chemistry and magnetic orientation: A has a $(PEI/PAA)_{15}$-$(PDDA/PSS)_{21}$-PDDA multilayer with north poles facing outward while B has a $(PEI/PAA)_{15}$-$(PDDA/PSS)_{21}$- PDDA/PAA multilayer with south poles facing outward. After self-propulsive motions, three isomers of ABA, triangled AAB, and lined AAB were observed. With magnetic forces only, local capillary adjustments were lacking to avoid the formations of AAB isomers. Therefore, the additive effect of magnetic/capillary forces was necessary to realize the self-sorting mechanism.
